# Antifatigue Activity of Liquid Cultured* Tricholoma matsutake* Mycelium Partially via Regulation of Antioxidant Pathway in Mouse

**DOI:** 10.1155/2015/562345

**Published:** 2015-11-30

**Authors:** Quan Li, Yanzhen Wang, Guangsheng Cai, Fange Kong, Xiaohan Wang, Yang Liu, Chuanbin Yang, Di Wang, Lirong Teng

**Affiliations:** ^1^School of Life Sciences, Jilin University, Changchun 130012, China; ^2^Zhuhai College of Jilin University, Jilin University, Guangzhou 519041, China; ^3^School of Chinese Medicine, Hong Kong Baptist University, Kowloon, Hong Kong

## Abstract

*Tricholoma matsutake* has been popular as food and biopharmaceutical materials in Asian countries for its various pharmacological activities. The present study aims to analyze the antifatigue effects on enhancing exercise performance of* Tricholoma matsutake* fruit body (ABM) and liquid cultured mycelia (TM) in mouse model. Two-week* Tricholoma matsutake* treatment significantly enhances the exercise performance in weight-loaded swimming, rotating rod, and forced running test. In TM- and ABM-treated mice, some factors were observed at 60 min after swimming compared with nontreated mice, such as the increased levels of adenosine triphosphate (ATP), antioxidative enzymes, and glycogen and the reduced levels of malondialdehyde and reactive oxygen species in muscle, liver, and/or serum. Further data obtained from western blot show that CM and ABM have strongly enhanced the activation of 5′-AMP-activated protein kinase (AMPK), and the expressions of peroxisome proliferator have activated receptor *γ* coactivator-1*α* (PGC-1*α*) and phosphofructokinase-1 (PFK-1) in liver. Our data suggest that both* Tricholoma matsutake* fruit body and liquid cultured mycelia possess antifatigue effects related to AMPK-linked antioxidative pathway. The information uncovered in our study may serve as a valuable resource for further identification and provide experimental evidence for clinical trials of* Tricholoma matsutake* as an effective agent against fatigue related diseases.

## 1. Introduction

Regular exercise has been confirmed to protect and alleviate various diseases; however, strenuous sports are responsible for the accumulation of reactive oxygen and lipid peroxides, which damage organ tissues and further lead to fatigue [1, 2]. Fatigue, caused by intense pressure from physical and mental work [3], affects more than 20% of people worldwide. Sleep disturbance, muscle pain, headache, and impaired concentration always appear in patients suffering from chronic fatigue. Repairing the damage occurring in organs and prompting elimination of metabolites accumulation during exercise are required for its recovery. In molecular level, the activation of 5′-AMP-activated protein kinase (AMPK) inhibits the overproduction of reactive oxygen species (ROS) [4] and suppresses anabolic ATP-consuming [5]. On the other hand, enzymatic and nonenzymatic antioxidants protect tissues from exercise-induced oxidative damage [6] and further reduce metabolite production and physical fatigue [7]. Intense exercise causes an imbalance between the body's oxidation and antioxidation systems; thus overproducing reactive oxide species (ROS) and malondialdehyde (MDA) is responsible for fatigue symptoms [8, 9]. The enhanced activities of superoxide dismutase (SOD) and glutathione peroxidase (GSH-Px) are observed combining with the antifatigue process [10].

Fatigue is becoming a serious public health problem, and what is worse current therapeutic regimen is far away from meeting the needs of patients. Since nutrient supplementation positively enhances exercise capacity, researches attempt to seek antifatigue herbs to accelerate eliminating fatigue in human beings [11].* Herba Rhodiolae*, commonly used for hypoxia cure by the Tibetan people [12], also enhances physical ability [13].* Astragalus membranaceus* has been confirmed to improve exercise performance and reduce exercise-caused accumulation of the byproducts in blood [14].* Cordyceps sinensis* improves animal exercise endurance via regulating glycogen breakdown and fatty acid oxidation which are accommodated by AMPK phosphorylation [15].

*Tricholoma matsutake*, which is an ectomycorrhizal symbiotic mushroom, has been popular as food and biopharmaceutical materials in Asian countries [16]. The immunomodulatory activities and anticancer effects of* Tricholoma matsutake* have been well established worldwide [17]. Various polysaccharides have been reported, which are separated from* Tricholoma matsutake* fruit body with antioxidant activity [18], antitumor effect [19], and immune regulation property [20]. However, wild fruiting body resources are scarce, and to cultivate the fruit bodies via artificial culture medium is limited. Submerged fermentation has been verified as an efficient way to produce mycelia and bioactive metabolites for fungus in amount of research groups [21, 22]. In our research group, submerged fermentation has been successfully applied to obtain mycelia of* Paecilomyces tenuipes* [23],* Marasmius androsaceus*, and* Irpex lacteus* [24]. One study shows that beta-glucan separated from liquid cultured mycelia of* Tricholoma matsutake* exerts immunostimulating activities via multiple signaling pathways linked to neural factor-kappa B activation [25].

To our knowledge, the antifatigue activities of both* Tricholoma matsutake* fruit body and liquid cultured mycelia have not been reported yet. Our present study aims to investigate the related biological activates of* Tricholoma matsutake* via mouse model. Antioxidant enzyme activities in serum and organs and AMPK activation in liver were assayed in order to further analyze its underlying mechanism.

## 2. Materials and Methods

### 2.1. *Tricholoma matsutake* Preparation

*Tricholoma matsutake* (CGMCC5.793) was cultured in a hundred-liter full-automatic fermentor (BaoXing Bioscience company, Shanghai, China) by using a defined liquid medium containing: 20 g/L sucrose, 10 g/L peptone, 10 g/L yeast extract powder, 0.5 g/L MgSO_4_·7H_2_O, 0.5 g/L KH_2_PO_4_·3H_2_O, and 0.2 g/L Vitamin B1. The fermentation conditions were as follows: loading volume was 70 L/100 L, initial pH was 6.5, rotate speed was 150 rpm, ventilation volume was 200 L/h, culture temperature was 26°C, inoculum age was 3 days, and inoculum size was 5%. The collected* Tricholoma matsutake* mycelium (TM) was freeze-dried, and it was given directly to experimental mice. All the chemical reagents used in the submerged fermentation were obtained from Sigma-Aldrich, USA.

### 2.2. Antifatigue Capacity Analysis in Mouse

The experimental animal protocol used in the study was approved by Institution of Animal Ethics Committee in Jilin University. Kunming (KM) mice (6 weeks, 18–22 g; *n* = 20/group; equal numbers of males and females; purchased from Norman Bethune University of Medical Science, Jilin University, Jilin, China) (SCXK(JI)-2011-0003) were put on a 12 h light/dark cycle (lights on 07:00–19:00) at 23 ± 1°C with water and food available ad libitum. After one-week adaptation, the experimental mice were divided into five groups randomly and orally treated with physiological saline (control group), 0.4 g/kg of* Tricholoma matsutake* body fruit micropowder (purchased from Japan) (ABM), and TM powder at doses of 0.3 g/kg, 1.0 g/kg, and 2.0 g/kg once a day for two weeks continuously. After two hours since the last administration, following experiments were performed.

#### 2.2.1. Weight-Loaded Swimming Test

Experimental mice were loaded with lead wire of 15% bodyweight and forced to swim in water of 22 ± 1°C temperature and 30 cm depth. The time from the beginning to the point at which mice failed to return to the water surface within 15 s was recorded as exhaustion time.

#### 2.2.2. Rotating Rod Test

Three independent trainings on turning device at a speed of 15 rpm for 1 min were applied before format detection. During the rotating rod test, the total duration of experimental mice on turning device at 20 rpm was recorded.

#### 2.2.3. Forced Running Test

Three independent trainings on a treadmill at a set speed of 15 rpm for 1 min were performed to all the mice. After practices, animals were put on the treadmill with a set speed of 20 rpm, and the total running time was recorded to evaluate their performance before exhaustion, which was identified as five-time consecutive shocks by the electrode.

### 2.3. Measurement of Physiological Indices Related to Fatigue

Kunming mice (6 weeks, 18–22 g; *n* = 20/group; equal numbers of males and females) were orally treated with physiological saline, 0.4 g/kg of* Tricholoma matsutake* body fruit powder, and TM powder at doses of 0.3 g/kg, 1.0 g/kg, and 2.0 g/kg once a day for two weeks continuously. After four hours since the last administration, blood samples were collected from caudal vein in all mice. The serum levels of ATP, MDA, SOD, and GSH-Px were determined according to the procedures provided by assay kits.

After 24-hour rest, the experimental mice were forced to swim in water with 22 ± 1°C temperature and 30 cm depth for 60 min. Blood samples were collected from caudal vein after 10 min recess. Further, liver and muscle of sacrificed mice were dissected, and they were homogenized in double distilled water after wash three times in ice-cold physiological saline solution. The levels of ATP, SOD, GSH-Px, ROS, and MDA in liver, muscle, and/or serum were detected. The concentration of glycogen in both muscle and liver was measured. All the assay kits were purchased from Nanjing Biotechnology Co. Ltd., Nanjing, China.

### 2.4. Western Blot Analysis

A part of liver tissue of each experimental mouse was homogenized in eight volumes of radioimmunoprecipitation assay (RIPA) buffer (Sigma-Aldrich, USA) which contains 1 mM Phenylmethanesulfonyl fluoride (PMSF) and 1x protease inhibitor cocktail (Sigma-Aldrich, USA). The protein concentration was detected by Bradford method, and 30 *μ*g of sample was separated by 10% SDS-PAGE and then transferred onto a nitrocellulose membrane (0.45 *μ*m, Bio Basic, Inc.) with the Mini-Protean two-gel electrophoresis system (Bio-Rad, USA). The blocked membranes with target strips were incubated with primary antibodies at 4°C overnight at dilution of 1 : 1000 as follows: phosphor-AMPK (P-AMPK), total-AMPK (T-AMPK), peroxisome proliferator activated receptor *γ* coactivator-1*α* (PGC-1*α*), phosphofructokinase-1 (PFK-1), and glyceraldehyde-3-phosphate dehydrogenase (GAPDH) (Cell Signaling Technology, Beverly, MA), followed by blotting with horseradish peroxidase-conjugated secondary antibodies diluted 1 : 2000 (Santa Cruz, USA). After visualizing with ECL detection system (GE Healthcare, UK), the intensity of target bands were quantified by using ImageJ.

### 2.5. Statistical Analysis

All data were expressed as mean ± SEM. A one-way analysis of variance (ANOVA) was used to detect statistical significance followed by post hoc multiple comparisons (Dunn's test) by using SPSS 16.0 software (IBM corporation, Armonk, USA). A *P* value < 0.05 was considered to be statistically significant.

## 3. Results

### 3.1. The Enhancing Exercise Endurance Ability of* Tricholoma matsutake*

**Figure 1:**
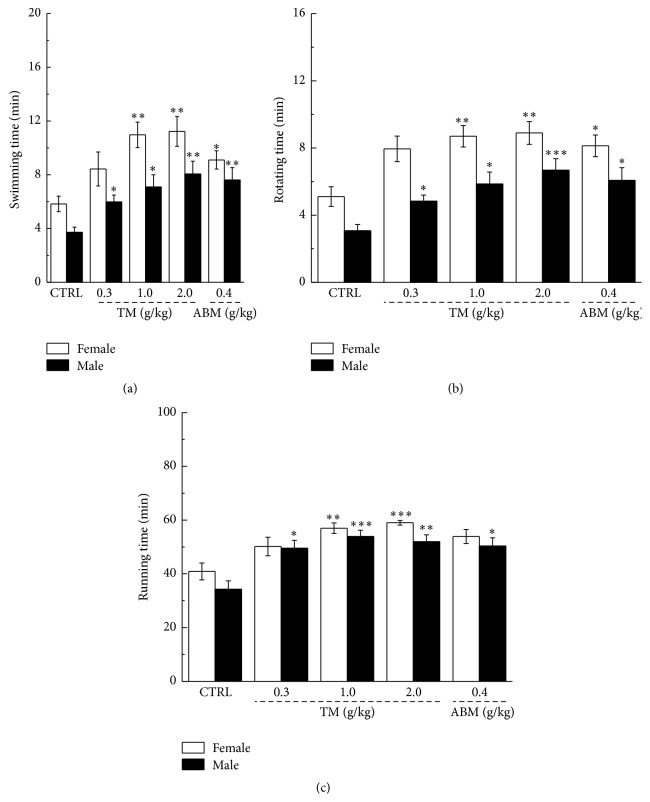
Weight-loaded swimming test (a), rotating rod test (b), and forced running test (c) were performed to verify the antifatigue effects of* Tricholoma matsutake* in mouse model. Data were expressed as mean ± SEM (*n* = 10) and analyzed by using a one-way ANOVA followed by Dunn's test. ^*∗*^ *P* < 0.05, ^*∗∗*^ *P* < 0.01, and ^*∗∗∗*^ *P* < 0.001 versus control group.

Behavior tests are commonly applied to evaluate the antifatigue performance of drugs [26, 27]; therefore, weight-loaded swimming, rotating rod, and forced running test were taken in the present experiment. Compared to nontreated mice, both TM and ABM have significantly enhanced the exercise endurance indicated by the longer movement duration in all three behavior tests (*P* < 0.05, Figure 1). 2.0 g/kg of TM administration improved nearly twofold of residence time of both female and male mice in all tests (*P* < 0.01, Figure 1). It seems that TM possesses more sensitive effects on athletic ability of female mice.

### 3.2. The Regulation Effects of* Tricholoma matsutake* on ATP and Glycogen

Compared to nontreated mice, no significant changes of serum levels of ATP were observed after 14-day TM and ABM administration (Table 1).

**Figure 2:**
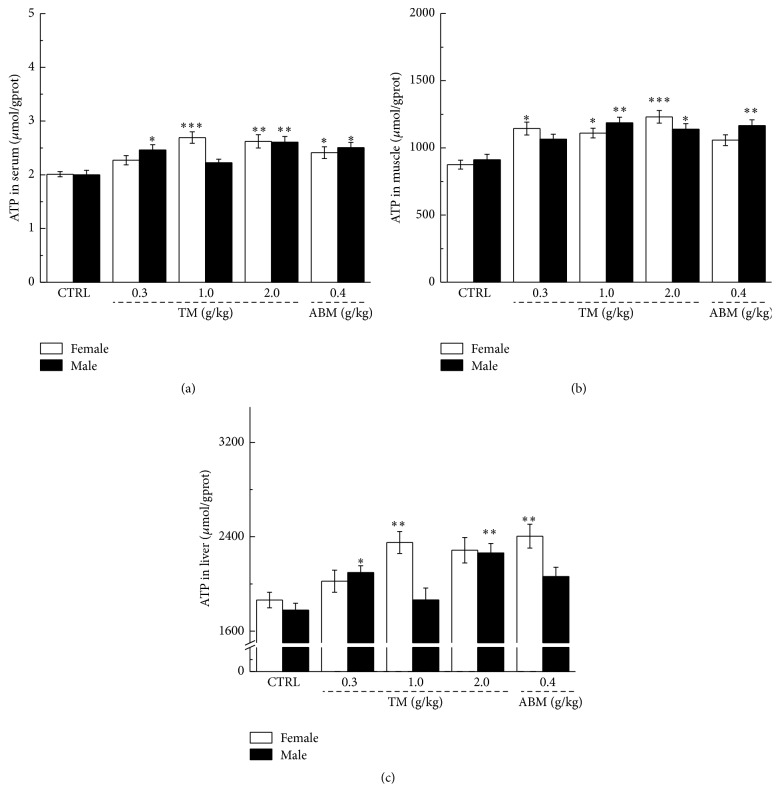
At the end of two-week administration, the levels of ATP in serum (a), muscle (b), and liver (c) in male and female mice were analyzed after 60 min of swimming. Data were expressed as mean ± SEM (*n* = 10) and analyzed by using a one-way ANOVA. ^*∗*^ *P* < 0.05, ^*∗∗*^ *P* < 0.01, and ^*∗∗∗*^ *P* < 0.001 versus control group.

ATP, as a direct energy source for normal physiological activity, shortage leads to a variety of physiological dysfunction [28]. Compared with nontreated mice, and similar to ABM, TM dose-dependently enhanced ATP concentration in serum, muscle, and liver after 60 min of swimming, and up to 30% enhancement was noted in both male and female mice (*P* < 0.01, Figure 2).

**Figure 3:**
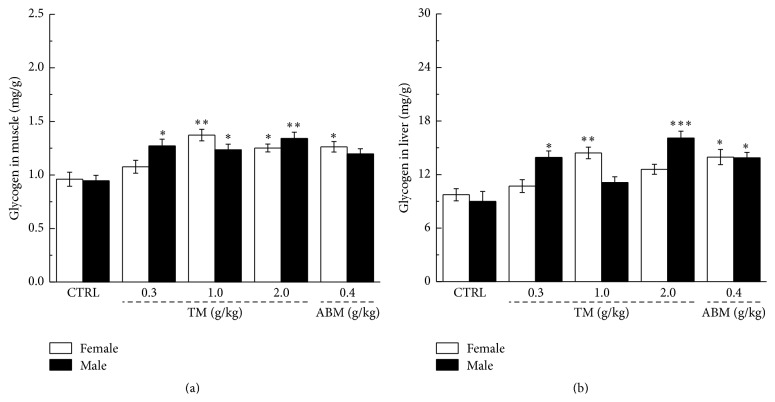
Two-week* Tricholoma matsutake* and ABM administration strikingly enhanced the levels of glycogen in both muscle (a) and liver (b) of exercise fatigue mouse. Data were expressed as mean ± SEM (*n* = 10) and analyzed by using a one-way ANOVA followed by Dunn's test. ^*∗*^ *P* < 0.05, ^*∗∗*^ *P* < 0.01, and ^*∗∗∗*^ *P* < 0.001 versus control group.

Glycogen is the primary intracellular storable form of glucose, and its level in liver and skeletal muscles directly reflects fatigue symptoms [29]. After 60 min exercise, compared with nontreated mice, two-week TM treatment at a dose of 2.0 mg/kg has enhanced 35.6% and 28.5% muscle glycogen levels in male and female mice, respectively (*P* < 0.05, Figure 3(a)). In liver tissue, TM has increased to 48.1% and 52.5% glycogen levels in female and male mice, respectively (*P* < 0.05, Figure 3(b)). The similar improvement activities on glycogen concentration in both muscle and liver were also observed in ABM-treated mice (*P* < 0.05, Figure 3).

### 3.3. The Antioxidant Effects of* Tricholoma matsutake*

Antioxidant enzymes, such as SOD and GSH-Px, are responsible for cell damage and muscle fatigue. MDA is one of the end products resulting from degradation of cell membrane by radicals [30]. Before swimming, no significant changes on levels of SOD, GSH-Px, and MDA in serum, muscle, and liver were observed in both TM- and ABM-treated mice (Table 1).

Compared to nontreated group, in both female and male mice, TM significantly enhanced the activities of SOD and GSH-Px and reduced the levels of MDA in serum, muscle, and liver after 60 min swimming (*P* < 0.05, Table 2). Similarly, ABM displayed positive regulatory effects on levels of SOD, GSH-Px, and MDA in serum muscle and liver of exercised male or female mice (*P* < 0.05, Table 2). The reduced ROS levels of muscle and liver were observed in TM- and ABM-treated mice after 60 min excise (*P* < 0.05, Table 2).

### 3.4. The Regulation Effects of* Tricholoma matsutake* on Protein Expression in Liver

**Figure 4:**
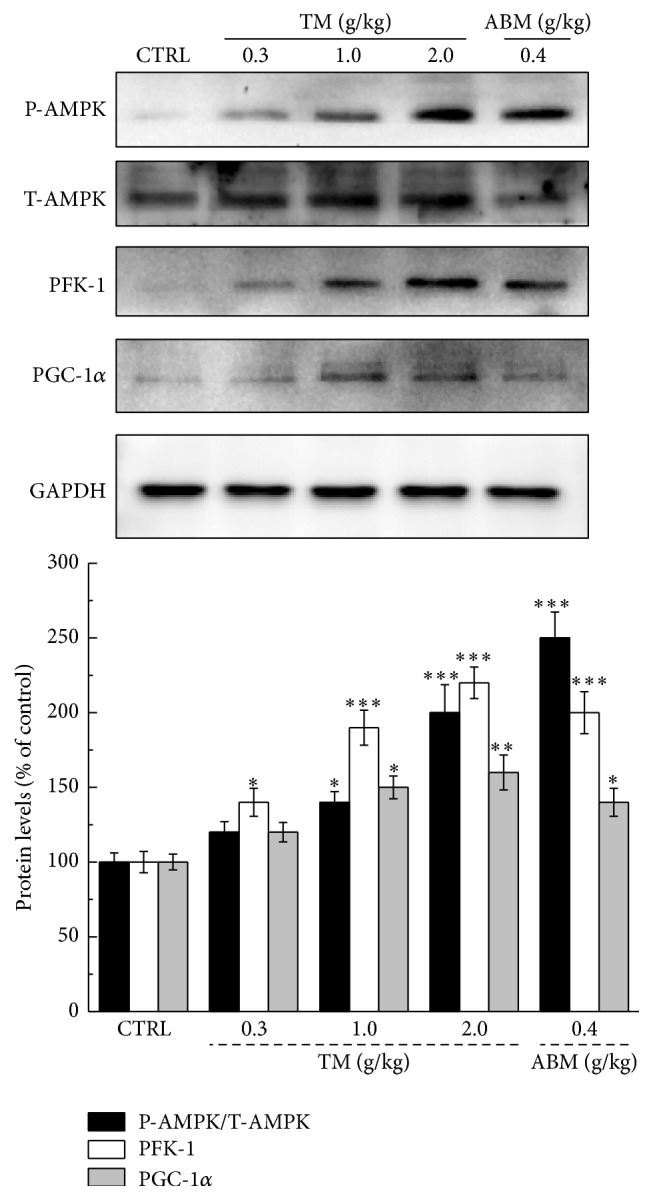
At the end of two-week* Tricholoma matsutake* and ABM administration, a 60 min swimming was performed. After exercise, the levels of P-AMPK, PFK-1, and PGC-1*α* in liver tissue were detected by western blot. The intensity of target bands was quantified by using ImageJ. Quantification data of P-AMPK, PFK-1, and PGC-1*α* were normalized by T-AMPK and GAPDH and expressed as a percent of that from corresponding control mice. Data are expressed as mean ± SEM (*n* = 4). ^*∗*^ *P* < 0.05, ^*∗∗*^ *P* < 0.01, and ^*∗∗∗*^ *P* < 0.001 versus control group.

The expressions of P-AMPK, T-AMPK, PFK-1, and PGC-1*α* were detected to further analyze the preliminary molecular mechanisms under TM-mediated antifatigue effects. After 60 min swimming, over twofold enhancement of P-AMPK expression was observed in liver tissue of TM- or ABM-treated mice (*P* < 0.001, Figure 4). Furthermore, TM dose-dependently enhanced the expressions of PFK-1 and PGC-1*α* in the liver of exercised mice, and the similar results were noted in ABM-treated group (*P* < 0.05, Figure 4).

## 4. Discussion

Herbs have become a valuable reservoir for antifatigue agents selection [31]. In our group, the polysaccharides-enriched* Cordyceps militaris* extract exhibiting exercise enhancement activity has been successfully demonstrated via modulation AMPK-linked pathway [32]. The present study aims to investigate the antifatigue effects of* Tricholoma matsutake* liquid cultured mycelia and fruit body in mouse model. The enhanced exercise endurance of TM- and ABM-treated mice in weigh-loaded swimming, forced running, and rotating rod test revealed the antifatigue activities of* Tricholoma matsutake*. The enhanced levels of ATP and glycogen in serum, muscle, and/or liver of treated mice have contributed to TM-mediate fatigue recovery. ATP, known as a rapid energy source, can be influenced by levels of muscle [H^+^] and myofibrillar ATPase during exercise [33]. However, the half-life of ATP is <1 second, and it makes glycogen become an indirect energy source for ATP synthesis. Long-term endurance exercise, which is related to muscle mitochondria dysfunction, results in a reduction of muscle glycogen depletion [34]. The regulatory effect of PGC-1*α* on mitochondrial biogenesis has been well reported, and exercises have influenced the expression of PGC-1*α* [35]. PGC-1*α* is involved in exercise-induced downregulation of the expression of glycogenolytic and glycolytic enzymes [34]. Another study reports that fatigue and exhaustion are viewed as a multicomponent biochemical process related to PFK-1 linked glycolytic pathway [36]. Although the enhanced expression of both PGC-1*α* and PFK-1 were observed in the liver of TM- and ABM-treated mice after 60 min exercise, it is hard to conclude that they are involved in the antifatigue effects. Based on our data, the enhanced ATP and glycogen levels in serum and organs by TM and ABM contribute to improve exercise endurance.

The regulatory effects on ROS, MDA, SOD, and GSH-Px in organs and/or serum of TM and ABM in mice after 60 min exercise indicated the important role of oxidative system in preventing exercise-induced fatigue. The accumulation of oxygen free radicals is one of the risk factors which are responsible for oxidative stress and muscle fatigue [37], and they can be cleared by antioxidant enzymes. Antioxidants can successfully prevent or reduce oxidative stress and further improve exercise performance [38]. Proteins, separated from* Panax quinquefolium*, improve behavioural alterations linked to chronic fatigue via inhibition oxidative damage [10]. Meanwhile,* Cordyceps militaris* displays antifatigue property via scavenging ROS and enhancing activities of SOD and GSH-Px [32]. MDA is recognized as the end product of lipid peroxidation, which can be used to assess the degree of the lipid peroxidation for free radical [39]. SOD prevents lipid peroxidation via catalyzing the conversion of superoxide into hydrogen peroxide and oxygen [37]. GSH forms a formidable defense with glutathione peroxidase to prevent lipid peroxides [40]. All these work together to prevent lipid peroxidation and further protect cells from oxidative injury via suppressing hyperlevel of MDA and ROS and enhancing SOD and GSH-Px activities, which are involved in TM- and ABM-mediated antifatigue effect on enhancing exercise endurance.

AMPK serves as the key factor on regulation of glucose and lipid metabolism [41] and helps to maintain the levels of ATP in various conditions [41]. The activated AMPK via falling cellular energy status enhanced ATP generation, whilst inhibiting ATP consumption [42]. AICAR, an AMPK agonist, significantly enhanced running endurance in animals [43]. On the other hand, except for the fatigue situation, during oxidative stress, the activated AMPK successfully promoted cell survival by inhibiting free radical accumulation [44]. Liraglutide reduced oxidative stress by the alteration of AMPK/SREBP1 pathway in Raw264.7 cells [45]. AMPK contributed to the antioxidant activity via regulating the levels of SOD and GSH [46, 47]. Both TM and ABM improved the activities of AMPK by enhancing the expression of its phosphorylated form in liver of 60 min exercised mice. Collectively, the antifatigue effects of* Tricholoma matsutake* may be related to the alteration of AMPK-linked antioxidant pathway.

Although the regulatory effects of TM and ABM on male and female mice were monitored during the experiments, we failed to conclude the different performances on animals of different genders. In the separated experiment, we found that the modulation of sex hormones contribute to the sex differences in response to* Cordyceps militaris*-mediated antifatigue property [32]. Our further study will focus on the regulatory effects of* Tricholoma matsutake* on sex hormones and the relationship between them and antifatigue activities.

In conclusion, our data indicate that TM and ABM elevate the endurance capacity at least in part via activating AMPK-linked antioxidant pathway, which provides experimental evidence in supporting the clinical use of* Tricholoma matsutake* as an effective agent against fatigue related diseases.

## Figures and Tables

**Table 1:** The effects of* Tricholoma matsutake* on fatigue and oxidant related factors in serum of mice before swimming.

	Female					Male				
	CTRL	TM (g/kg)			ABM (g/kg)	CTRL	TM (g/kg)			ABM (g/kg)
		0.3	1	2	0.4		0.3	1	2	0.4
ATP (nmol/mL)	2.5 ± 0.1	2.6 ± 0.1	3.1 ± 0.1	2.9 ± 0.1	2.7 ± 0.1	2.6 ± 0.1	2.7 ± 0.1	2.9 ± 0.1	2.8 ± 0.1	2.7 ± 0.1
MDA (nmol/mL)	11.4 ± 0.4	11.2 ± 0.4	10.8 ± 0.3	11.3 ± 0.3	11.3 ± 0.3	12.7 ± 0.4	12.1 ± 0.8	11.7 ± 0.6	11.9 ± 0.3	12.3 ± 0.3
SOD (U/mL)	90.4 ± 5.3	93.0 ± 3.1	101.1 ± 3.3	99.6 ± 3.1	99.7 ± 4.8	93.2 ± 4.4	96.2 ± 3.3	93.4 ± 3.1	100.4 ± 2.5	97.8 ± 5.1
GSH-Px (*μ*mol/mL)	156.7 ± 10.6	172.1 ± 8.3	171.0 ± 10.1	176.2 ± 10.0	174.1 ± 9.4	159.0 ± 16.7	167.6 ± 10.2	168.9 ± 12.3	169.7 ± 7.6	171.5 ± 14.4

Values were expressed as mean ± SEM (*n* = 10).

**Table 2:** The antioxidant effects of *Tricholoma matsutake* in exercise fatigue mice.

	Female					Male				
	CTRL	TM (g/kg)			ABM (g/kg)	CTRL	TM (g/kg)			ABM (g/kg)
		0.3	1	2	0.4		0.3	1	2	0.4
Serum										
MDA (nmol/mL)	15.5 ± 0.4	14.3 ± 0.7	13.9 ± 0.3^*∗*^	13.4 ± 0.4^*∗∗*^	13.7 ± 0.4^*∗*^	17.2 ± 0.5	14.2 ± 0.5^*∗*^	14.8 ± 0.5^*∗*^	14.7 ± 0.4^*∗∗*^	14.7 ± 0.4^*∗∗*^
SOD (U/mL)	105.8 ± 4.0	120.7 ± 2.1^*∗*^	133.2 ± 6.5^*∗∗*^	139.2 ± 5.5	141.4 ± 5.9^*∗∗*^	106.7 ± 3.0	118.9 ± 4.9	141.3 ± 3.5^*∗∗*^	136.1 ± 3.2^*∗∗*^	137.8 ± 3.3^*∗∗∗*^
GSH-Px (*μ*mol/mL)	357.9 ± 8.4	390.3 ± 4.8^*∗*^	427.5 ± 10.4^*∗∗∗*^	418.1 ± 15.9^*∗*^	404.5 ± 8.3^*∗∗*^	340.1 ± 11.8	417.2 ± 12.5^*∗∗*^	397.2 ± 12.1^*∗*^	415.1 ± 14.3^*∗∗*^	401.3 ± 9.1^*∗∗*^
Muscle										
MDA (nmol/mgprot)	63.1 ± 4.1	51.04 ± 3.1	46.7 ± 2.1^*∗*^	41.3 ± 3.4^*∗∗*^	48.7 ± 3.4	65.3 ± 2.4	53.1 ± 2.9	49.5 ± 3.5^*∗*^	44.6 ± 3.9^*∗∗*^	50.8 ± 3.2^*∗*^
SOD (U/mgprot)	87.7 ± 3.1	106.9 ± 5.4	111.5 ± 5.7^*∗*^	130.9 ± 4.4^*∗∗∗*^	114.1 ± 5.1^*∗∗*^	86.6 ± 4.5	104.5 ± 6.8	111.5 ± 5.3^*∗*^	127.8 ± 6.0^*∗∗∗*^	114.1 ± 4.6^*∗∗*^
GSH-Px (*μ*mol/gprot)	369.2 ± 29.2	469.6 ± 48.2	559.8 ± 47.6	550.4 ± 38.8^*∗∗*^	514.9 ± 32.1^*∗*^	371.2 ± 27.8	499.5 ± 42.5	591.6 ± 45.9^*∗∗*^	558.6 ± 46.7^*∗*^	538.9 ± 42.5
ROS (FI/gprot)	15300.7 ± 516.3	14335.0 ± 386.2	12461.6 ± 378.2^*∗∗*^	13037.7 ± 527.4^*∗*^	14277.3 ± 548.6	14294.1 ± 401.6	13532.2 ± 591.9	14396.8 ± 464.7	12162.4 ± 689.7^*∗*^	11669.1 ± 690.7^*∗*^
Liver										
MDA (nmol/mgprot)	12.2 ± 0.7	9.8 ± 0.5	8.7 ± 0.4^*∗∗*^	8.8 ± 0.5^*∗*^	9.7 ± 0.6	11.8 ± 0.4	9.6 ± 0.7	9.3 ± 0.6	8.8 ± 0.7^*∗*^	8.5 ± 0.7^*∗*^
SOD (U/mgprot)	270.5 ± 17.8	369.8 ± 18.6^*∗*^	387.5 ± 22.7^*∗*^	303.9 ± 18.3	317.9 ± 17.6	240.9 ± 19.2	316.7 ± 16.5	365.4 ± 22.6^*∗∗*^	296.5 ± 15.6	335.9 ± 17.4^*∗*^
GSH-Px (*μ*mol/gprot)	1013.5 ± 50.9	1244.7 ± 67.5	1264.0 ± 99.3	1361.5 ± 60.4^*∗∗*^	1371.9 ± 74.1^*∗*^	929.2 ± 32.1	1169.0 ± 55.1^*∗*^	1098.0 ± 44.4	1365.9 ± 91.8^*∗∗*^	1100.8 ± 77.9
ROS (FI/gprot)	1516.2 ± 53.9	1494.2 ± 33.6	1209.1 ± 55.3^*∗∗*^	1356.8 ± 46.9	1360.7 ± 30.1	1606.6 ± 75.9	1446.9 ± 46.8	1360.7 ± 62.6	1278.4 ± 51.4^*∗*^	1286.3 ± 52.7^*∗*^

After 60 min swimming, the oxidative stress factors in serum, muscle, and liver of all experimental mice were detected. Data were expressed as mean ± SEM (*n* = 10). ^*∗*^ *P* < 0.05, ^*∗∗*^ *P* < 0.01, and ^*∗∗∗*^ *P* < 0.001 versus control group.
